# Materials and interfaces properties optimization for high-efficient and more stable RbGeI_3_ perovskite solar cells: optoelectrical modelling

**DOI:** 10.1038/s41598-023-42471-w

**Published:** 2023-09-19

**Authors:** Soulye Samaki, Fridolin Tchangnwa Nya, Guy Maurel Dzifack Kenfack, Amel Laref

**Affiliations:** 1 Materials Science Laboratory, Department of Physics, Faculty of Science, University of Maroua, P.O. Box 814, Maroua, Cameroon; 2 Department of Physics and Astronomy, College of Science, King Saud University, 11451 Riyadh, Saudi Arabia

**Keywords:** Renewable energy, Devices for energy harvesting

## Abstract

In this research work, we investigated the effects of a broad set of materials properties and external operating parameters on the opto-electrical output of a hybrid RbGeI_3_-based perovskite solar cell (PSC) as a means of enhancing its performance. We first performed a judicious numerical modelling of the reference cell with the following structure FTO/TiO_2_/RbGeI_3_/Spiro-OMeTAD/Ag, with data retrieved from the experiment. SCAPS program enables to model the device, considering charge carriers transport governing equations. Investigations are directed on addressing the current challenges that include thinner, less environmentally harmful, cost-effectiveness, and more stable solar devices over time. Analysis of the effects of different hole transport material (HTM) on current–voltage (J-V) and external quantum efficiency (QE) characteristics, helps to identify CuI as an ideal HTM. Optimal cell output were achieved by investigating the effects of metal contact work function, defect states, RbGeI_3_ thickness, light transmission/reflection at the front/back contact, as well as operating temperature. As a result, efficiency increased significantly from 10.11 to 18.10%, and fill factor that represents a stability indicator, increased from 63.68 to 76.95%. Moreover, an optimum open-circuit voltage Voc = 0.70 V and a high short-circuit current density of Jsc = 33.51 mA/cm^2^ were recorded. An additional study on the capture cross-section of charge carriers (${\sigma }_{n,p}$) on PV characteristics, enabled to achieve a power conversion efficiency (PCE) of 29.71% and FF of 88% at a value of ${\sigma }_{n,p}$ selected to be 10^–22^ cm^2^. This contribution aims at designing and producing thinner, more efficient, more stable and more environmentally clean and economically viable PSCs.

## Introduction

Nowadays, solar cells are an essential common resource in the modern era in which the consumption of energy is growing owing to the massive development of infrastructure in our society. Numerous research axes are currently being investigated to improve their efficiency to meet energy demand. Consequently, solar cell production increases twice a year on average. Solar cell structures are composed of an absorber or active layer, where the majority of charge carriers in the cell are photogenerated through light absorption. Following the absorber layer, there are the electrons and holes transport layers that transport previously generated charge carriers towards cathode and anode. These layers are critical as they mainly contribute to solar device performance. The hybrid Perovskite solar cells (PSCs), which are based on any organic–inorganic active material complex following the formula **AMX**_**3**_^1,2^, where **X** is a halide ion, **M** is a tiny divalent metal cation, and **A** is a cation of organic/inorganic nature, analogous to calcium titanate CaTiO_3_; they are positioning themselves as a serious competitor (PCE ~ 24%) to silicon technology (PCE ~ 27%)^3^. The photoactive properties of perovskites exhibit excellent carrier mobilities (10–200 cm^2^V^–2^ s^–1^), negligible dipolar binding energies (0.3 eV), long carrier diffusion lengths (> 1 µm)^4^, excellent carrier lifetimes (270 ns), dramatic defect densities tolerance (10^16^ cm^–3^), robust bipolar transport properties, and a high dielectric constant^4^. In addition to its tunable band gap characteristics (1.24–1.7 eV)^5,6^, simple solution process application techniques, low-price, and smooth availability, it is a rapidly-growing PV technology. Nonetheless, relevant challenges remain to be addressed. These factors includes but are not limited to, the toxicity issue of certain materials within the structure, phase stability of the material during the various transition processes, long-term stability, thickness-performance trade-off, degree of crystallinity obtained by reducing bulk and interfaces defect states and grain boundaries, robustness to external temperature variations when operating, light collection optimization through the engineering of surfaces and interfaces, rapid transport and optimal collection of photogenerated charge carriers. The present work not only aims to address these concerns, but is also a step towards designing more efficient and stable solar devices. Following on from this work, the RbGeI_3_ perovskite complex will be employed as the active material. An optoelectrical modelling approach is adopted, by utilizing SCAPS-1D numerical environment to perform the analysis of some relevant PV outputs, such as current–voltage, quantum efficiency and energy band characteristics. We first optimised the architecture and certain properties of the reference cell^7^ in order to improve the very low fill factor, which is a valuable indicator of device stability^8^. To achieve this, we study the effects of active layer thickness, bulk and interfaces defects states density, surface and interfaces light properties through light transmission/reflection ratios at front/back contact, and the impact of front/back metal contact work function. Indeed, an effective charge carrier collection process can be achieved when the work function of the front contact material is equivalent to or slightly exceeding that of the conduction band of the electron transport materials, allowing a better alignment of the energy band alignment with the absorber. After optimizing the device structure, we investigate how to further improve the efficiency (PCE) and fill factor. Recalling that hole transport material (HTM) is reported as impacting the performance and the consistency of PSCs, we investigate on the replacement of the Spiro-OMeTAD HTM, herein denoted SPO. Even if SPO exhibits a relatively low electron recombination rate, which is beneficial for hole transport, its high cost hampers its large-scale production^2^. Alternative HTM such as CuI, Cu_2_O, CuSCN are prospected as a trade-off between cost-effectiveness, toxicity and performance. Although NiO enabled achieving good results in the reference work^7^, it is excluded in this work to address some environmental and health concerns^9–12^. As in most solution-processed semiconductors, trap density is significant in perovskites, and its effects are most evident under continuous excitation^13^. As evidenced by the typical charge carrier lifetime of perovskites, which ranges from several nanoseconds to a few microseconds, trapping has a negative effect on optoelectronic devices despite its low capture cross-section. This is why, we supplemented a study of the impact of charge carriers capture cross-section on the performance of an optimized designed RbGeI_3_-based PSC device, as a way of further enhancing efficiency and stability.

## Materials and methods

### Methods

Optoelectrical modelling and PV characteristics calculations are performed by means of SCAPS software^14–16^, a reliable program recently employed by a fairly broad PV community to model perovskite thin-film solar devices^1,17–20^ and the results achieved are fairly consistent with experiments. Owing to the asymmetric designs of solar cells, the work function offset between the anode and cathode, together with the alignment of energy levels at the interfaces, induce a built-in electric field normal to the interfaces. This facilitates the capture of charge carriers at electrode connections by easing drifting motion of charge carriers^21^. Neutral defects with Gaussian distribution and characteristic energy set at 0.1 eV are assumed to exist at mid-band gap level. A detailed study of the influence of defects density (${N}_{t}$) and charge carriers capture cross sections (${\sigma }_{n,p}$) at the interface and bulk level of the active layer can be performed using the Shockley–Read–Hall (SRH) recombination model on cell performance. The SRH recombination model can be described as follows^22^:

1 $${R}^{SRH}=\frac{{\upsilon }_{th}{\sigma }_{n,p}{N}_{t}\left[np-{n}_{i}^{2}\right]}{n+p+2{n}_{i}\mathrm{cosh}\left(\frac{{E}_{t}-{E}_{i}}{kT}\right)}.$$

The capture cross-section for electrons or holes depends on their lifetime (${\tau }_{n,p}$) before they recombine into exciton as follows^23^:

2 $${\sigma }_{n,p}=\frac{1}{{\tau }_{n,p}{\upsilon }_{th}{N}_{t}}\stackrel{this means }{\to }{ \tau }_{n,p}=\frac{1}{{\sigma }_{n,p}{\upsilon }_{th }{N}_{t}},$$

*n* and *p* represent electron and hole densities, respectively, under the non-equilibrium condition. ${n}_{i}$ indicates the intrinsic density, ${E}_{i}$ denotes the intrinsic energy level, and ${E}_{t}$ stands for the energy level of the trap defects. ${\upsilon }_{th}$ represents the thermal velocity of electrons and holes. Solar cell performance has been monitored using current–voltage (*J–V*), and quantum efficiency (*QE*) characteristics, short-circuit current density (${J}_{SC}$), open-circuit voltage (${V}_{OC}$), fill factor (FF), and power conversion efficiency (PCE). In the absence of internal resistance effects, the current–voltage characteristic can be formulated as follows^24^:

3 $$i={i}_{ph}-{i}_{0}\left[exp\left(\frac{qV}{\beta kT}\right)-1\right],$$

where ${i}_{0}$, indicates the dark saturation current, as provided by^25^:

4 $${i}_{0}=q\left(\frac{{D}_{n}{n}_{i}^{2}}{{L}_{n}{N}_{A}}+\frac{{D}_{p}{n}_{i}^{2}}{{L}_{p}{N}_{D}}\right).$$

The open-circuit voltage (Voc) can be estimated via the following equation:

5 $${V}_{OC}=\frac{akt}{q}\mathrm{ln}\left(\frac{{i}_{ph}}{{i}_{0}}+1\right),$$

where *a* is a factor and (*kt/q*) is a thermal voltage. The external quantum efficiency (EQE) is ruled by the radiative and non-radiative recombination taking place in the major exciton formation zone, and is expressed as^26^:

6 $${EQE}_{EL}=\frac{{J}_{0, rad-bi}}{{J}_{0, rad-bi}+{J}_{0, nr-bi}+{J}_{0, nr-trap}},$$

where ${J}_{0, rad-bi}$ represents the background current density resulting from the radiative portion of molecular recombination, ${J}_{0, nr-bi}$ denotes the background current density coming from the nonradiative portion of molecular recombination, and ${J}_{0, nr-trap}$ represents the background current density associated with non-radiative trap-assisted recombination.

### Choosing materials, phase stability, preparation and synthesis of RbGeI_3_

In the general formula **AMX**_**3**_**,** as stated in the introduction, one of the key challenges in using organic–inorganic perovskite materials as active layers in solar devices is their long-term operating stability. This also raises the concern of the phase stability within the material during the preparation, synthesis and growth. Indeed, low phase stability is likely to hamper the large-scale production^27^. In general, phase instability within organic–inorganic perovskites crystals is mainly attributed to the organic component. This also supports the choice of rubidium (Rb) as the inorganic cation instead of the conventional methylammonium, ethyl-ammonium or formamidinium. Saliba et al.^28^ also advocated the incorporation of rubidium cations as an efficient way of significantly improving PSC stability. For toxicity concern, the divalent germanium cation (Ge2 +), has drawn interest as a suitable alternative to the toxic and unstable lead (Pb). Indeed, it exhibits a smaller atomic diameter than Sn2 + and Pb2 + , better conductivity, and the ability to build stable perovskite structures. For the stability issue, Krishnamoorthy et al.^29^ synthesized three AGeI3 (A = Cs, CH_3_NH_3_ or HC(NH_2_)_2_) halide perovskite materials and found out that they are stable up to 150 °C mainly attributed to Ge material. Meng et al.^30^ supported achieving best performance using Ge-based PSC. Moreover, Deepthi and Sebastian^31^ conducted an ab-initio DFT determination of structural, mechanical, optoelectronic, thermoelectric and thermodynamic properties of RbGeI_3_ and concluded that it has all the characteristics required as a stable absorber material. Further, Mitro et al.^32^ investigated on the electronic phase transition and mechanical properties of RbGeI_3_ and came out with the statement that it exhibits a ductile and anisotropic nature. According to Parrey et al.^33^, RbGeI_3_ does not exhibit any magnetic phase and is stable in ferromagnetic environments as a result of its structure, magneto-electronic and elastic properties analysis. However, the structural stability study through the phonon dispersion spectrum^34^ shows the presence of imaginary modes indicating an unstable structure in the cubic phase. This structure which can undergo a phase transition to orthorhombic phase at high temperature (> 200 °C). RbGeI_3_ can be prepared following the solid-state reaction scheme described by the work of Satta et al.^27^ for CsPbI_3_ since they have the same structure. High-quality RbGeI_3_ layer with uniform morphology and superior crystallinity, can be obtained employing one of the following deposition techniques: one-step and two-step chemical solution deposition, vapour-assisted solution deposition, and co-evaporation deposition.

### Device modelling

**Figure 1:**
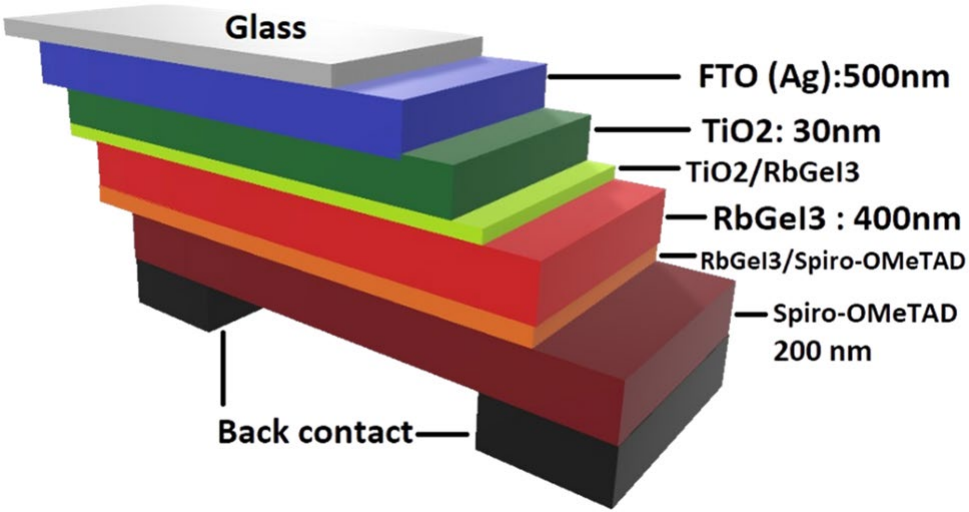
RbGeI_3_-based device structure.

This study utilizes the structure as reference cell according to Pindolia et al.^7^ work, which has the following structure: FTO/TiO_2_/ RbGeI_3_/Spiro-OMeTAD/Ag, as depicted in Fig. 1. TiO_2_ is the ETL, RbGeI_3_ is the active material, Spiro-OMeTAD (SPO) is the HTL and Ag acts as anode. It is noteworthy that in Pindolia et al. obtained the following performances with NiO and CuI as HTM, PCE 9.89% and 12.81%, and FF 64.28% and 12.21%, respectively. Their se results revealed that, CuI exhibited the best PCE, but why was the fill factor so poor? Firstly, we excluded NiO from this work for the reasons outlined in the introduction section. This is why we will conduct this study by replacing Spiro-OMeTAD HTM with the following materials: CuI, Cu_2_O and CuSCN. Our second hypothesis is that certain significant parameters were not taken into account during modelling, leading to the poor fill factor. As a continuation of their improvements, we will carry out more realistic simulations by modelling the two interfaces ETL/RbGeI_3_ and RbGeI_3_/HTM. Indeed, both sides interfaces located between the active layer and ETM and HTM are areas of high properties discontinuity, and failing to take it into consideration may lead to inconsistent results. Moreover, bulk and interface s defects states are assumed, to consider both bulk and interfaces recombination phenomena. We then carry out optimization calculations that incorporate numerical models of front/back contact effects on light transmission/reflection. By considering the evolution of J-V and QE characteristics, we also investigate the effects of defects states density, active layer thickness, and metal back contact work function^7^. All simulations are carried out considering an external illumination of 1000 Wm^–2^ at AM 1.5G and an operating temperature of 300 K. The thermal velocity of electrons and holes is set to 107 cm/s. Defects are considered neutral by assuming a gaussian distribution. The values of materials, HTMs, interfaces and defects properties are gathered in Tables 1, 2 and 3.

**Table 1:** SCAPS input parameters for the device configuration.

Properties	Spiro-OMeTAD	RbGel_3_	TiO_2_	FTO
Thickness (nm)	200	400	30	500
${E}_{g}$(eV)	2.88	1.31	3.2	3.2
$\chi$(eV)	2.05	3.9	4	4.4
${\varepsilon }_{r}$(eV)	3	23.01	100	9
${N}_{V}$(cm^–3^)	2.5E20	2.8E19	2E20	1.8E19
${N}_{C}$(cm^–3^)	2.5E20	1.4E19	1E21	2.2E18
${\mu }_{e}$(cm^2^/Vs)	2.1E-3	28.6	6E-3	20
${\mu }_{p}$(cm^2^/Vs)	2.6E-3	27.3	6E-3	10
${N}_{D}$(cm^–3^)	0	1E9	5.06E19	1E19
${N}_{A}$(cm^–3^)	1E18	1E9	0	0
${N}_{T}$(cm^–3^)	1E15	1E15	1E15	1E14

**Table 2:** SCAPS input parameters of different HTMs.

Properties	Cu_2_O	CuI	CuSCN
Thickness (nm)	200	200	200
${E}_{g }$(eV)	2.17	2.98	3.4
$\chi$ (eV)	3	2.1	1.7
${\varepsilon }_{r}$ (eV)	7.5	6.5	10
${N}_{V}$ (cm^–3^)	1.1E19	1E19	1.8E18
${N}_{C}$ (cm^–3^)	2E18	2.8E19	2.2E19
${\mu }_{e}$ (cm^2^/Vs)	200	100	100
${\mu }_{p}$ (cm^2^/Vs)	80	43.9	25
${N}_{D}$ (cm^–3^)	0	0	0
${N}_{A}$ (cm^–3^)	2E19	2E19	2E19
${N}_{T }$(cm^–3^)	1E15	1E15	1E14

**Table 3:** Interface states parameters.

Interface state	TiO_2_/RbGeI_3_	RbGeI_3_/Spiro-OMeTAD
Defect type	Neutral	Neutral
Capture cross: electron section ${\upsigma }_{\mathrm{e}}$	5E-16	5E-16
Capture cross: hole section ${\upsigma }_{\mathrm{h}}$	5E-16	5E-16
Energetic distribution type	Gaussian	Gaussian
reference for defect energy level Et	Above the highest EV	Above the highest EV
Energy with respect to Reference (eV)	0.6	0.6
Defect density ${\mathrm{N}}_{\mathrm{t}}$ $({\mathrm{cm}}^{-3})$	1E16	1E16

## Results and discussion

### Effects of active layer thickness

**Figure 2:**
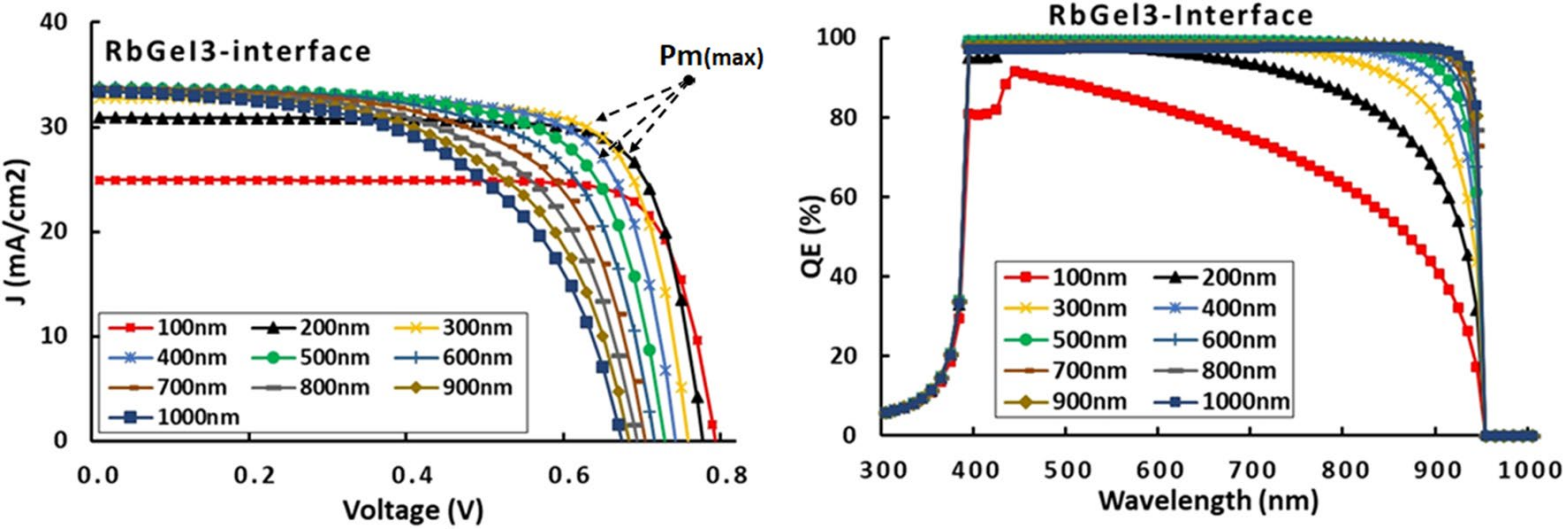
J-V and QE characteristics as a function of RbGeI_3_ thickness considering RbGeI_3_ interface.

The perovskite cells belong to the thin film devices, with a thickness of active layer generally around 500nm^35^. They are known for producing good yield even at very thin thickness. As depiected in Fig. 2, this is confirmed by the progression of J-V curves. As apparent, the most efficient devices are recorded between 200 and 600 nm active layer thickness according to their J-V curves that exhibit the highest mean power ${P}_{m}$ (${P}_{m}={I}_{m}\times {V}_{m}$). As a result of the longer stay of the incident light within the material when an absorber layer is thicker than an active layer, the optical path length of the incident light within the active layer will be longer^36^. It also increases the probability of photons being absorbed and generates more charge carriers. This obviously results in an improvement in cell conversion efficiency. In contrast, as the depth of the active layer exceeds these values, the J-V characteristics are found to be lower, indicating less efficient devices. The reduced thickness has the positive side of reducing the deposition time and therefore reducing the cost of the process. It is found that a thickness of 200 to 400nm is the optimum operating range.

### Effects of defects states density

**Figure 3:**
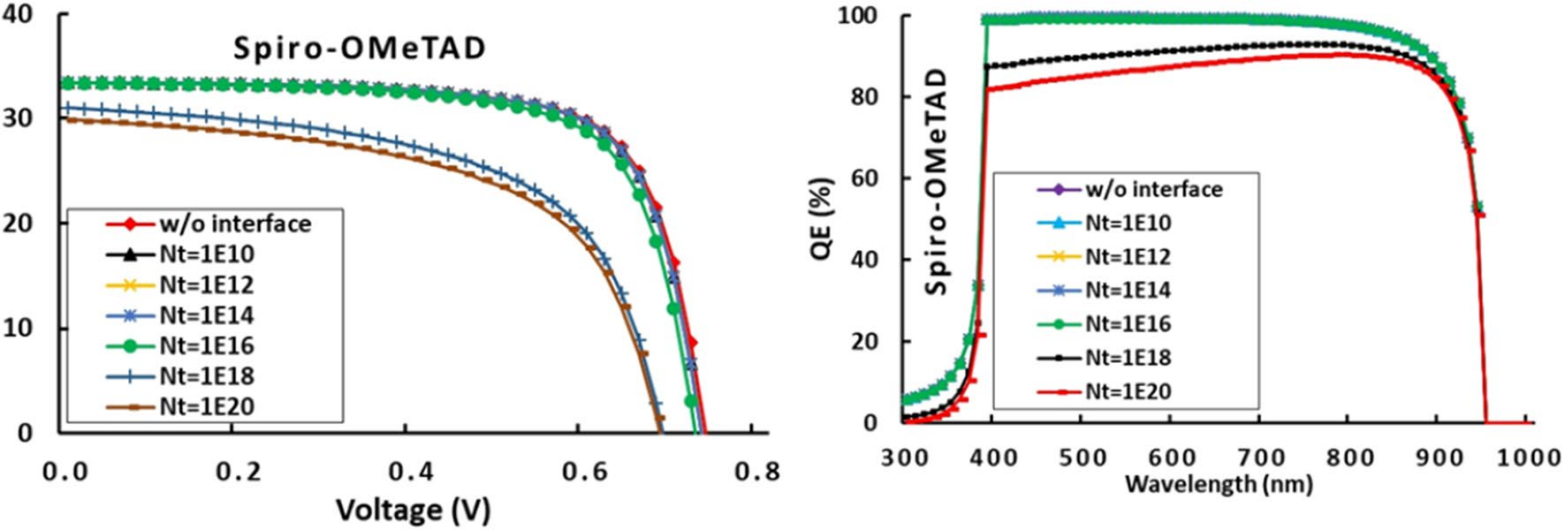
J-V and QE characteristics as a function of bulk defects states density Nt.

It is possible to categorize the defects states in PSCs either as interruptions or as impurities in the pristine crystal lattice. Since they appear during the deposition, material preparation, or growth processes, their presence in the structure is inevitable^35,37^. More realistic results can be obtained by modelling a device structure by considering their density in the volume or at the interface. In this work, defects states density (Nt) impacts on PSC performances are studied by varying Nt from 10^10^ to 10^20^ cm^−3^, which means from a closed to a pure device to a very contaminated material. As can be seen from Fig. 3, the external QE of the cell drops from nearly 96 to 80%. Therefore, 20% of the incident light is not absorbed by the structure. In fact, increasing Nt results in increasing deep trap sites for charge carriers at the levels of bulk and surface defects or at grain boundaries. This induces more bulk non-radiative charge recombination in the active layer of the PSCs. If defects are located inside the structure and materials with a density ranging between 10^10^ and 10^16^, the device can function efficiently. In addition to locating the range of tolerance of impurities in the active layer for efficient operation, this result is relevant for experimental work. Additionally, this study agrees well with Sunny et al.^38^. statement that obtained low defect densities devices, i.e. with Nt less than 10^14^ cm^−3^ in the experiment, is troublesome with the present manufacturing techniques, and reported, a defect density of 10^14^ cm^−3^ as an optimized value.

### Effects of light transmission/reflection at front/back contact on QE efficiency

**Figure 4:**
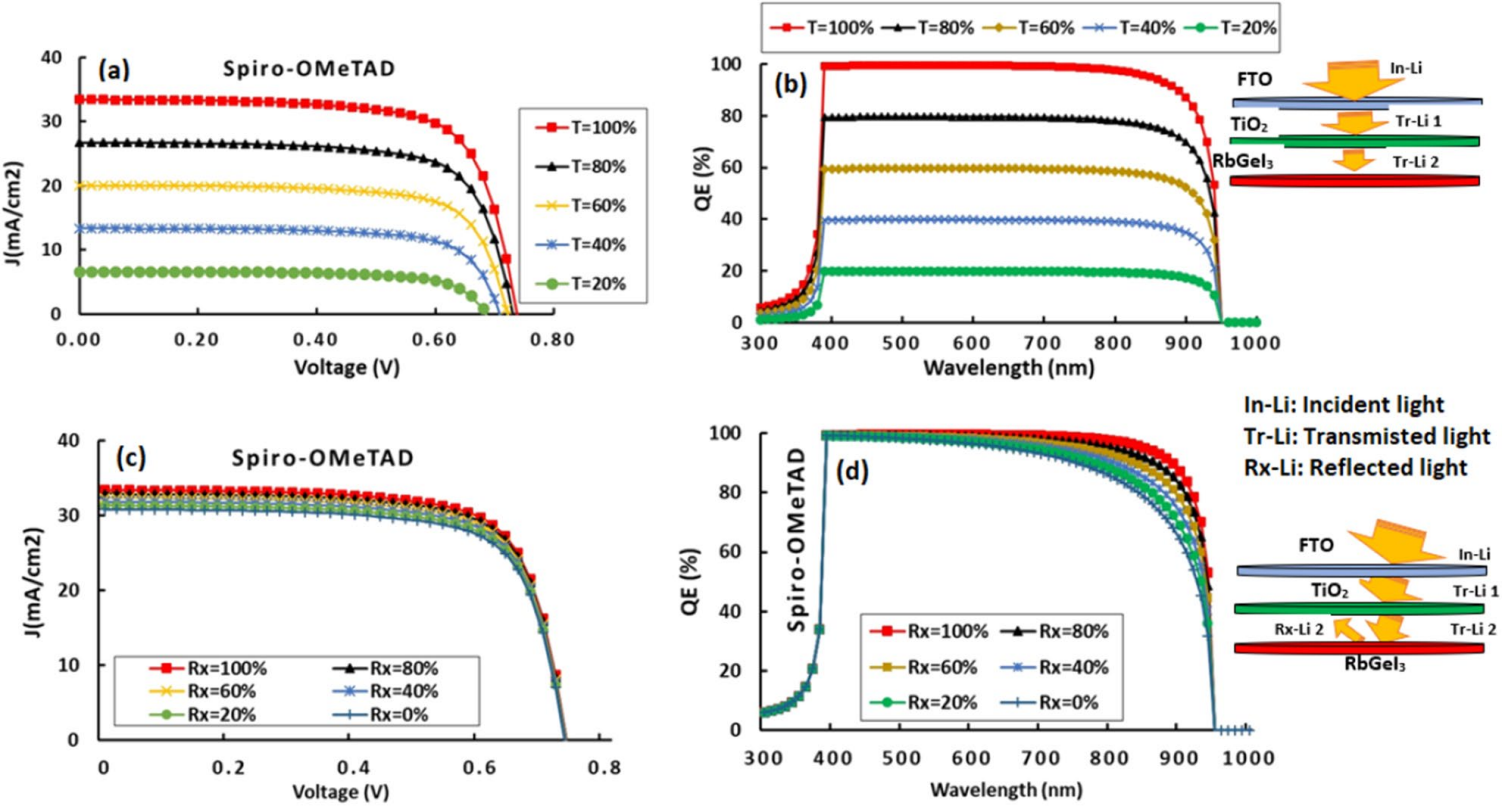
Effects of light transmission/reflection ratio at front/back contact on RbGeI_3_-based PSC performance.

Assuming total transmission of the incident light flux at the front contact or no light reflection at the inner layers including the rear contact, we are pretty sure to simulating an extremely unrealistic cell. Obviously, not all incident light can be fully transmitted into the active layer, a portion will be definitely lost due to absorption in the preceding layers i.e. FTO and TiO_2_. It is only possible to model light transmittance at the FTO that acts as the front contact using SCAPS. Figure 4a and b illustrate the trend of the J-V and QE characteristics of the cell as a function of the percentage of light transmittance through the front contact when it is varied from 20 to 100%. This feature doesn’t significantly impact Voc, and its value remains concentrated in the range [0.70; 0.75]. On the other hand, Jsc is severely impaired. As a result, its value drops from 34 to 0.6 mA/cm^2^ when T_x_ drops from 100 to 20%. In the same way, EQ drops from 97 to 20%. As the transmission rate is reduced, the number of photons reaching the transmission layer is decreased. During the absorption of these photons, the major charge carriers are generated, and therefore the current is generated.

As the active layer is quite thick (500 nm), the few photo-generated carriers must be recombined before they can reach the splitting interface. One strategy to compensate for the low light transmission of the window layer and the front contact might need to reduce the thickness of the active layer near the optimum range, as found between 200 and 400 nm in Sect. “Effects of active layer thickness”. Fortunately, the window and front contact layers have been designed with a gap high enough (3.2 eV) to allow a significant portion of the visible spectrum to flow into the active layer. The performance of a cell made from FTO and ETM materials that are not transparent enough will be poor.

Furthermore, the surface of each inner layer will reflect micro-beams of light, but what is the direct effect of this reflected light on the cell's performance? Once again, SCAPS can only reflection percentage at the back contact^16^. RbGeI_3_ traps a substantial amount of incident light with a band gap of 1.31 eV. When some of the light that escapes towards the back contact is reflected at its back contact surface, it is reabsorbed by the active layer, which directly boosts the absorption capacity of the active layer by generating additional charge carriers that enhance the photo-generated current, and therefore the current through the cell. As a matter of fact, it could only enhance the performance of the cell, particularly in terms of current. This explains the evolution of J-V and QE characteristics in Fig. 4c and d. Voc remains unchanged when the current evolves substantially from 30 to 34 mA/cm^2^ as R_x_ rises from 20 to 100%. Improved QE occurs in the spectral region of 700 to 1000 nm, which corresponds mainly to the part of the light that has not been absorbed during the first travel in the active layer. Due to the fact that it addresses the need to design solar devices with reflective surfaces at the back contact, this outcome is quite relevant^39^. In general, these results are in agreement with previous research^1^. This illustrates the importance of disposing of materials with a maximum transmittance rate that may allow the maximum of the solar spectrum to be transmitted.

### Effects of metal contact work function

**Figure 5:**
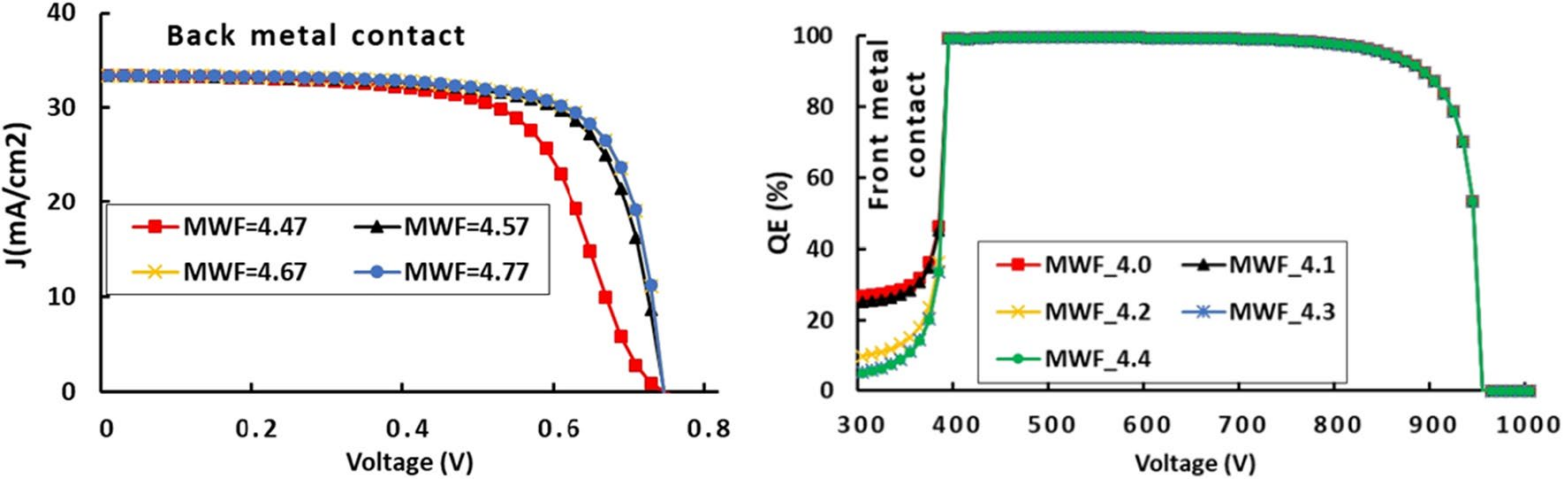
J-V and QE characteristics of RbGeI_3_-based PSC as a function of metal back/front contact work functions.

**Figure 6:**
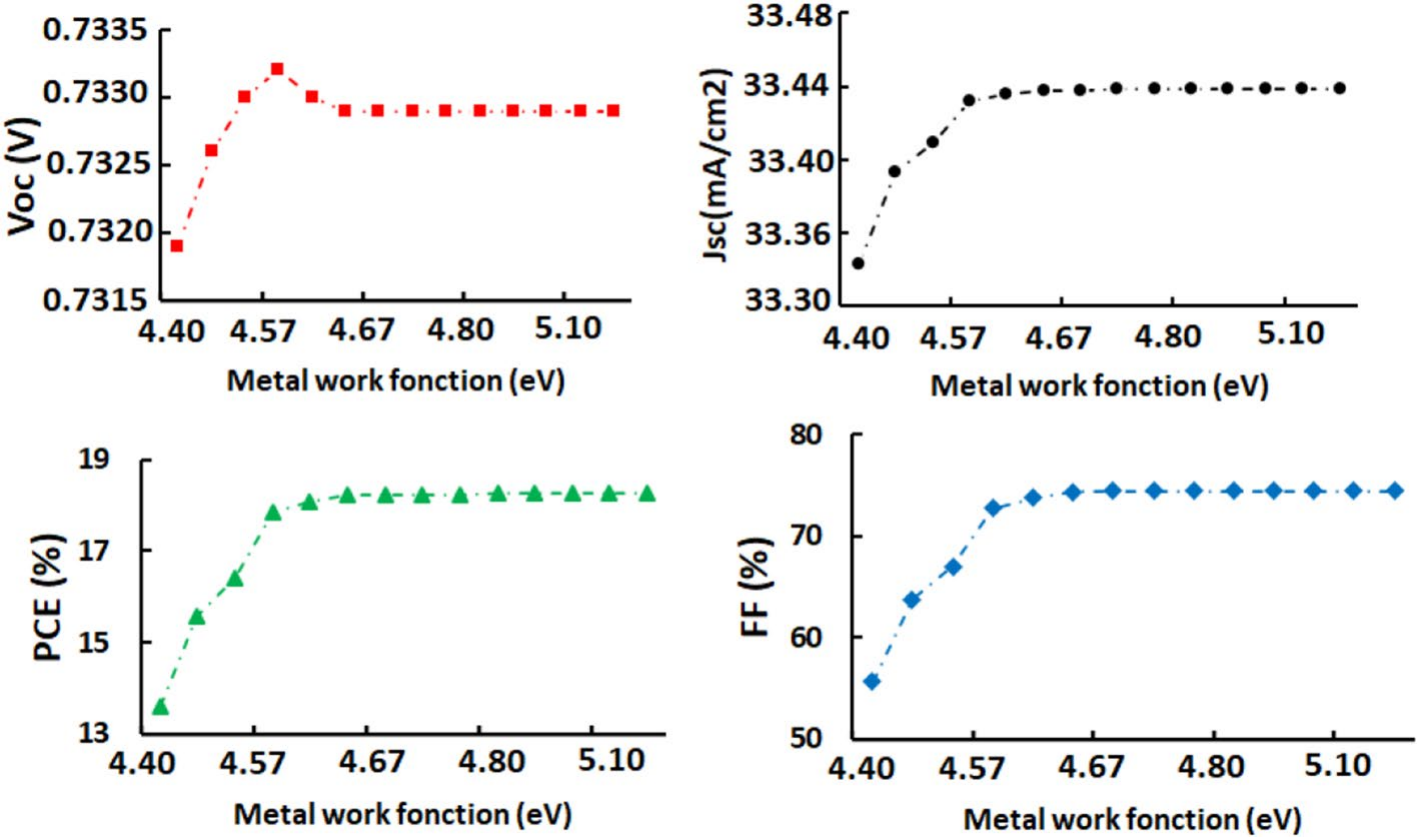
RbGeI_3_-based PSCs output characteristics as a function of metal back contact work functions.

At least one electrode in PSC devices must have a work function (WF) low enough to either inject electrons into or collect electrons from the conduction band. Back metal contacts should exhibit a higher work function to easily collect all holes dissociated at the RbGeI_3_/ETL interface. Moreover, the higher the WF of back contact, the more efficient is the collection of holes because they can easily migrate from HTL to highest energy level. However, the WF of the metal back contact should act as a barrier from electron. When carrying out the calculations, the optimum WF for metal back contact was found to be 4.77 eV. As depicted in Fig. 5, the highest J-V curves are obtained with WF ranging from 4.57 to 4.77 eV. Above that value, simulations produced steady results. This is perfectly highlighted in Fig. 6, since Voc, Jsc, PCE and FF all remain nearly constant once one crosses the threshold value of 4.77 eV for the work function of the metal back contact. A similar conclusion was made by Salem et al.^40^ who attributed the improved open-circuit voltage, and hence the greater efficiency of their PSC device to the built-in potential enhancement.

As opposed to the preceding one, the effects related to the work function of metal constituting the front contact follow a reverse logic. The cell material is responsible for effectively collecting that occur from the ETM after they have been dissociated at the ETL/RbGeI_3_ interface. The lower the work function, the more cost-effective the cell, since electrons reaching the latter with higher energy migrate more easily to lower energy levels. In right side of Fig. 5, there is a clear improvement in the (QE) characteristics mainly located at a very small portion of the visible spectrum concentrated between 300 and 400 nm. The optimum is reached for WF = 5.01 eV. Once this value is exceeded, the work function of metal front contact has little influenced by the output characteristics of the cell.

### Effects of HTM and energy band diagram of the optimized structure

**Figure 7:**
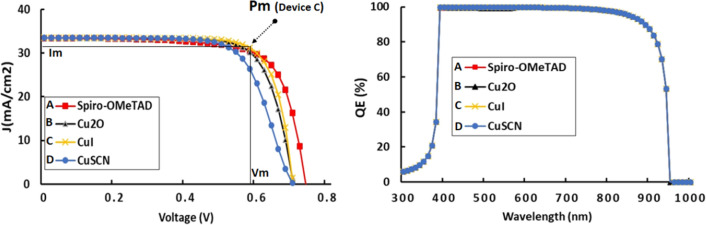
J-V and EQE characteristics for different HTMs.

In the solar architecture, the HTM fulfils two major functions: (1) providing an accessible energy level for the photo-generated holes within the active layer in order to facilitate their rapid transport to the rest of the circuit and to prevent their recombination (2) blocking the electrons that can be repelled by a high enough energy barrier. Since perovskite exhibits a P-type conductivity mostly provided by holes, the nature of the material used as HTM has a significant impact on the cell performance^41^. In selecting the right HTM, the following main features must be considered: the gap, the dielectric constant, the density of charge carriers, and the density of states in the valence band. There are quite a few studies on the impact of HTM on PV outputs^42,43^. As part of this section, a comparative study is conducted to assess the impact of incorporating different HTMs on J-V and EQ. The reference cell uses Spiro-OMeTAD and the other 03 cells incorporate Cu_2_O, CuI and CuSCN respectively, whose key properties values are gathered in Table 2. From Fig. 7, the structure that incorporates CuI as HTM exhibits the best performances as shown by its J-V curve, which registers the highest mean power. Moreover, as indicated in Table 4, the best performances evaluated in terms of power conversion efficiency (PCE = 18.10%) and fill factor (FF = 76.95%) are recorded with **Device C**. The hole mobility magnitude of 44 cm^2^/Vs is nearly 1000 times greater than that of Spiro-OMeTAD, which explains the enhanced flow of holes. Moreover, its dielectric constant (6.5 eV) is more than twice that of Spiro-OMeTAD, and it is well known that the higher E is, the weaker the strength between charge carriers in this material, explaining the high mobility of the holes perfectly. As HTM has a higher gap (2.98 eV) than spiro-OMeTAD (2.88 eV), plus the narrower range of light that reaches it, while it plays a less significant role in EQ, and it participates only slightly in the creation of charge carriers. This is why the EQ curves of the different structures are all overlapped.

**Table 4:** Comparative table of the performance of PSCs structures with different HTMs.

PSC structure	V_OC_ (V)	J_SC_ (mA/cm^2^)	FF (%)	PCE (%)
A: FTO/TiO_2_/RbGeI_3_/spiro-OMeTAD/Ag	0.73	33.43	72.49	17.88
B: FTO/TiO_2_/RbGeI_3_/Cu_2_O/Ag	0.70	33.46	74.70	17.58
C: FTO/TiO_2_/RbGeI_3_/CuI/Ag	0.70	33.51	76.95	18.10
D: FTO/TiO_2_/RbGeI_3_/CuSCN/Ag	0.70	33.50	69.59	16.37

**Figure 8:**
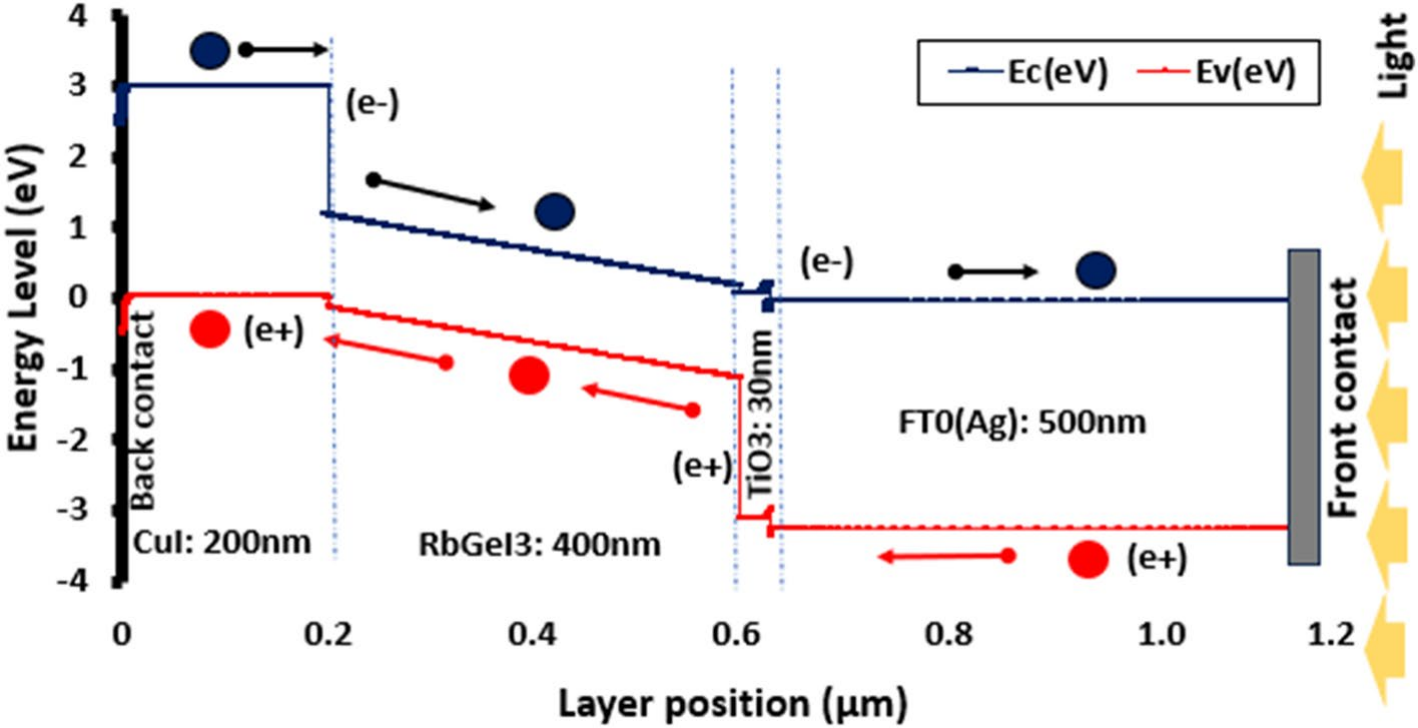
Energy band diagram illustration of FTO/TiO_2_/RbGeI_3_/CuI/Ag structure with charge carrier’s flow.

Charge carrier transport as depicted in Fig. 8 of the optimized device's energy level diagram, displays the transfer of the photo-generated electrons-holes across the device. Electron may easily migrate from higher energy bands to lower energy levels, such as CuI (E_C_ ~ 3 eV) to RbGeI_3_ (E_C_ ~ [0.5: 1.5 eV]), TiO_2_ (E_C_ ~ 0.5 eV), and lastly FTO (E_C_ ~ 0.2 eV). Similarly, holes transport is effective due to the valence levels configuration: the layer underneath the one with the highest valence energy for viable involvement in charge carrier photo-generation. The holes can freely migrate from FTO (E_V_ ~− 3.2 eV) to TiO_2_ (Ev ~− 3.1 eV), then to RbGeI_3_ (Ev ~ [− 1.2: − 0.1 eV]), and then to CuI (Ev ~ 0 eV). A moderate upward cleavage is detected at the TiO_2_/RbGeI_3_ interface, which, rather than being a degrading factor, inhibits superfluous photo-generated electrons and holes, from flowing through the conduction and valence bands, respectively, which may rapidly result in current saturation.

### Impacts of charge carriers capture cross-sections

**Figure 9:**
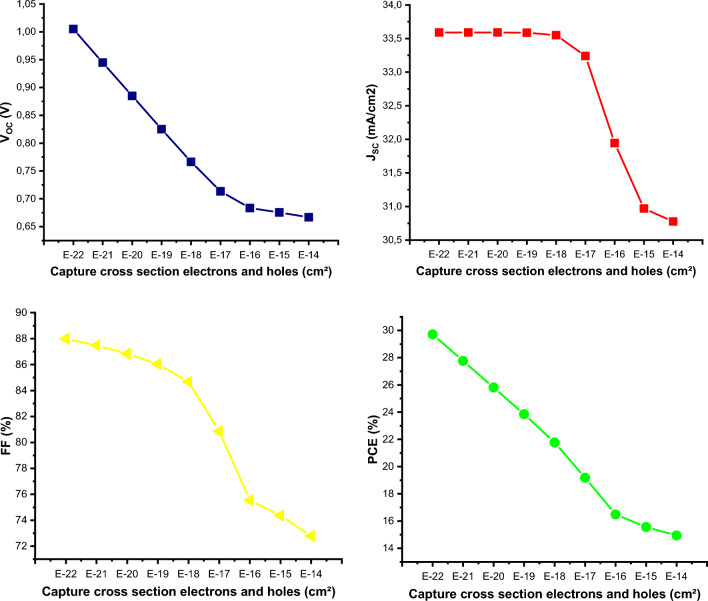
Effects of capture cross-section variation at active layer RbGeI_3_ and both the two interfaces RbGeI_3_/CuI and TiO_2_/RbGeI_3_ on output performances considering a defect density N_t_ = 10E^15^.

We conducted modelling in this section, by assuming that electrons and holes capture cross sections are equal, i.e. at each moment ${\sigma }_{n,p}={\sigma }_{n}={\sigma }_{p}$. The numerical analysis is conducted by considering only the variation of capture cross sections in the volume and at both the interfaces of RbGeI_3_/CuI and TiO_2_/RbGeI_3_. The capture cross-section measures the effective cross-section of the trap levels between the valence band and the conduction band that can capture photogenerated carriers reaching these sections at a given thermal velocity ${V}_{th}$. To be consistent with previous simulations results we considered a defect density value of 1E15 cm^–3^ in the volume and at the interfaces, and the simulations are carried out by varying ${\sigma }_{n,p}$ from 1E-14^44^ to 1E-22 cm^2^^6,45^. As it can be seen from Fig. 9, all PV output parameters are strongly affected by variation of ${\sigma }_{n,p}$. The smaller the ${\sigma }_{n,p}$, the more efficient the device is. According to Eq. (1) as ${\sigma }_{n,p}$ decreases SRH recombination current also decreases significantly, thus improving the open-circuit voltage (Voc), reducing the cell losses and thereby enhancing the performances of the device. Furthermore, keeping the defect density Nt constant, and remaining at constant thermal simulation conditions, i.e. ${V}_{th}={C}^{te}$, it follows from Eq. (2) that a reduction in ${\sigma }_{n,p}$ results in an increase in the lifetime of the charge carriers, and therefore an increase in their propensity to contribute to the photo-generation of current. By varying ${\sigma }_{n,p}$ from 1E-14 to 1E-22 cm^2^, the Voc augments from 0.66 to 1.00 V, while the short-circuit current density increased from 30.77 to 33.59 mA/cm^2^, which enables achieving the exceptional results with a PCE = 29.71% and an FF = 87.99%. This observation is fully confirmed by the statements of Arbia et al.^46^ who carried out a numerical analysis based on experimental results on a GaSb(p) /GaAlAsSb(p/n) /InAsSb(n) /GaSb(n) multijunction system. Their study examined the impact of defects at the GaAlAsSb/InAsSb interface, both donor and acceptor type defects, with high capture cross sections and low energy levels, and found that the conversion efficiency is affected by accumulation regime and depletion in the InAsSb layer. Ibrahim et al.^47^ also investigated the impact of carriers capture cross-section area at absorption layer on the performance of a CH_3_NH_3_PbI_3_ -based PSC by varying ${\sigma }_{n,p}$ from 2E-18 to 2E-10 cm^2^, and found that the PCE and FF increased from 1 to 13% and 30 to 75%, respectively. Sunny et al.^6^ achieved a conversion efficiency of 26.33% along with Voc of 0.98 V, Jsc of 31.93 mA/cm^2^, and an FF of 84.34% when performing their numerical simulations on 1.0 μm thickness of a PSC, and considering a capture cross-section ${\sigma }_{n,p}$=1E-19 cm^2^, of cell. Small charge carriers capture cross-sections can be obtained through an efficient passivation at the interface layer. In addition, fewer bulk defects density materials will be used and the difference in grain size will be reduced at the boundaries.

## Conclusion

The present study is based on a reference work on a perovskite solar cell with the following initial performances PCE of 10.11% and FF of 63.68%. In the aim of addressing current challenges in the industry, such as more stable, cost-efficient, and thinner cells, we conducted optimisation calculations by varying some of the material properties of the functional layers on the output characteristics. In order to determine the impact of variations in these parameters on the actual performance of a structure, the current–voltage (J-V) and quantum efficiency (QE) characteristics are excellent optoelectrical indicators. At the end of the different calculations, we recorded substantial improvements in the PCE, which was enhanced from 10.11 to 18.10%, and the fill factor, an excellent stability indicator, which was ameliorated from 63.68 to 76.95%. As a result of studying the effects of charge carriers capture cross-sections (${\sigma }_{n,p}$) on PV characteristics it enables to achieving both a PCE of 29.71% and a FF of 87.99%, using a device with ${\sigma }_{n,p}$=1E-22 cm^–2^. It is evident from this work that the optimization approach used is promising and has enabled to identify CuI as a future material for high efficiency and more stable perovskite solar cells on one hand, and manufacturing smaller charge carriers capture cross-sections obtained through efficient passivation, reduction in grain size differences at the boundaries and a lower defect density material on the other hand. We suggest the following architecture for future PSCs: FTO/TiO_2_/RbGeI_3_/CuI/Ag.

## Data Availability

The datasets used and/or analysed during the current study available from the corresponding author on reasonable request.
